# Sustainable safety practices and hazard management in the oil and gas industry: an HSE perspective

**DOI:** 10.3389/fpubh.2025.1611106

**Published:** 2025-08-06

**Authors:** Zara Jamil, Shahrina Nordin, Mohammad Miraj, Mazen Alqahtani, Riyaz Ahamad Shaik, Shamim Akhter, Ahmad Shahrul Nizam Isha

**Affiliations:** ^1^Department of Management and Social Sciences, Institute of Smart and Sustainable Living, University Teknologi PETRONAS, Bandar Seri Iskandar, Malaysia; ^2^Department of Physical Therapy and Health Rehabilitation, College of Applied Medical Sciences, Majmaah University, Al Majmaah, Saudi Arabia; ^3^Occupational Health and Safety, Diriyah Company, Ad Diriyah, Saudi Arabia; ^4^Department of Family and Community Medicine, College of Medicine Majmaah University, Majmaah, Saudi Arabia; ^5^Department of Information System Management, Stanton University, Garden Grove, CA, United States

**Keywords:** hazard identification and risk assessment, occupational health and safety, safety communication, safety performance, sustainable safety practices, safety knowledge, safety culture

## Abstract

Despite advancements in operational technologies, the oil and gas (O&G) industry continues to face safety lapses due to persistent challenges in occupational health and safety management (OHSM), hazard identification and risk assessment (HIRA), and safety communication (SC). This study aims to examine how OHSM, HIRA, and SC influence safety knowledge (SK) and safety performance (SP), with safety culture (SCULT) mediating these relationships. A novel framework, integrating technical, procedural, and cultural dimensions is proposed and empirically tested. Grounded in social exchange theory (SET), this framework is distinct in its dual focus on system-level safety practices and cultural mechanisms in high-risk environments. Malaysia’s downstream O&G sector was chosen due to its operational complexity, multicultural workforce, and elevated accident rates, making it a critical context for evaluating sustainable safety interventions. Data was collected from 350 employees from the operational department using stratified sampling across two major national oil companies PGB and MLNG. Partial least squares structural equation modeling (PLS-SEM) was employed to validate the model, demonstrating strong reliability and predictive relevance (SRMR = 0.064, AVE > 0.5). Notably, SC exhibited weak direct effects on SK and SP, but strong indirect effects via SCULT, suggesting that communication-based safety interventions are only effective when trust and cultural alignment are present. Behavioral outcomes such as proactive safety participation and cognitive outcomes such as hazard recognition were both positively influenced by a strong SCULT. This research offers practical strategies for industry stakeholders, including the adoption of a near-miss reporting system, behavior-based safety (BBS) training programs, and culturally adaptive communication audits. Policymakers are encouraged to embed cultural indicators within national safety audit frameworks and promote leadership accountability across organizational levels. The findings emphasize that achieving sustainable safety outcomes require more than structural compliance with culturally integrated safety systems.

## 1 Introduction

Sustainability, within high-risk industries is no longer confined to environmental stewardship and economic efficiency. It now necessitates equal emphasis on the social dimensions that are particularly related to the health and safety of workers. In the Malaysian oil and gas (O&G) sector, where operational complexity and hazard exposure are prevalent, the integration of occupational health and safety (OHS) practices into sustainability agendas has become imperative. Traditionally, the industry has emphasized economic metrics such as lifecycle cost and return on investment, alongside environmental targets like emissions control and resource efficiency (1). However, recent literature acknowledges that sustainable industrial development is unattainable without ensuring the physical, mental, and emotional well-being of the workforce (2, 3).

The downstream segment of Malaysia’s O&G industry which encompasses refining, processing, and distribution is especially prone to safety lapses due to the hazardous nature of its operations, aging infrastructure, and high workforce density. Although major legislative frameworks such as the 1984 Petroleum Act and 1994 Occupational Safety and Health Act (OSHA) have established safety standards, the sector continues to experience incident rates. For example, Malaysia recorded a 125% increase in O&G worker fatalities between 2009 and 2015, a trend that significantly contrasts with countries like the United States, where O&G-related fatalities account for approximately 18% of all industrial deaths (4). Hong Kong O&G sector has been cited as responsible for nearly 75% of worker fatalities, underscoring the severity of risks inherent to the industry. These trends suggest critical limitations in the effectiveness of conventional OSHMS in comprehensively addressing complex operational hazards. Moreover, macroeconomic stressors such as global financial downturns, COVID-19-related budget constraints, and energy market volatility have led many organizations to deprioritize safety investments in favor of cost containment. As a result, safety training programs, supervisory oversight, and communication systems are often underfunded or fragmented, especially in emerging economies. This reinforces the urgent need for resilient and culturally embedded safety frameworks capable of withstanding such external pressures. (1, 3, 5).

The selection of Malaysia’s downstream O&G sector for this study is deliberate. This segment represents a high-density, multi-ethnic, and operationally intense environment, often subject to both internal organizational challenges and external regulatory and economic fluctuations. Its strategic relevance in Malaysia’s national energy landscape, coupled with a rising concern over accident rates, makes it an ideal context for examining sustainable safety practices. Recent studies have pointed to the growing importance of safety culture (SCULT) and communication in shaping safety outcomes, yet several challenges persist. Safety communication (SC), although widely acknowledged as critical, lacks standardization in definition and implementation across regulatory bodies and organizational levels (6–8). This ambiguity hinders consistent enforcement and impairs the feedback loop required for responsive hazard management. Similarly, the concept of SCULT though recognized as a basis of sustainable safety practices remains difficult to operationalize, especially when disaggregated from organizational climate and leadership engagement. The industry continues to struggle with inadequate hazard identification and risk assessment (HIRA) practices, fragmented communication structures, and a disconnect between formalized safety procedures and employee behaviors on the ground (9–11).

While various studies have independently examined the roles of OHSM, HIRA, and SC in improving safety performance (SP). Yet, their combined influence and interaction, especially through the mediating mechanism of SCULT, are not well understood. Furthermore, limited empirical evidence exists on how these constructs affect safety knowledge (SK), a critical component for employee preparedness, hazard recognition, and safety decision-making. This is particularly relevant in Malaysian downstream O&G sites. These are sites where hierarchical communication patterns, multinational workforce dynamics, and complex site operations can dilute safety priorities and hinder knowledge retention.

To address this knowledge gap, the present study proposes and empirically tests a structural model that captures the interrelationships among OHSM, HIRA, SC, SCULT, SK, and SP. By elucidating these relationships, the study aims to provide valuable insights for targeted interventions that enhance sustainable safety practices (SSP) and prevent accidents. Grounded in social exchange theory (SET), this research posits that a reciprocal relationship exists between employees and their organization: when organizations invest in transparent communication, participatory safety practices, and effective HIRA protocols, employees are more likely to respond with increased safety compliance, knowledge sharing, and proactive behavior. SET provides a theoretical lens through which the motivational and behavioral aspects of workplace safety can be better understood, particularly in relation to trust, fairness, and mutual accountability.

This study’s primary objective is to explore the multidimensional dynamics of sustainable safety practices in the Malaysian downstream O&G industry. Using partial least squares structural equation modeling (PLS-SEM) and survey data from 350 workers from operational department, this research:

- Investigate how OHSM, HIRA, and SC affect SCULT.
- Examine the role of SCULT in mediating their impact on SP and SK.
- Identifies barriers and enablers of effective SC and hazard management.
- Provides actionable insights for developing targeted interventions to enhance sustainable safety outcomes.

This study aims to contribute to the growing body of occupational health and safety research by offering a robust, evidence-based framework that supports policy formulation, capacity building, and operational excellence in high-risk industrial settings. It also sets a precedent for evaluating SCULT not as a standalone construct, but as a dynamic mediator that reflects the depth and quality of organizational safety practices.

These research understanding can be applied not just in Malaysia’s downstream O&G industry, but also in other high-risk sectors globally. It can also support the development of practical strategies to improve safety within O&G companies. These strategies can then be disseminated and adapted for use in other industries facing similar challenges. Thus, the proposed framework directly contributes to achieving a more sustainable O&G industry. By promoting a safety-conscious workforce and preventing accidents, the proposed approach can minimize environmental risks and ensure the well-being of workers essential elements for long-term industry sustainability.

The rest of the paper is organized as follows: Section 2 provides the background to identify the knowledge gap and develop hypotheses. Section 3 details the research method used, including the quantitative approach and data analysis techniques. Section 4 presents the study’s findings and explores their significance for safety practices in the O&G industry. Finally, Section 5 concludes the paper by summarizing the key takeaways, limitations, and future directions.

## 2 Background

**Figure 1:**
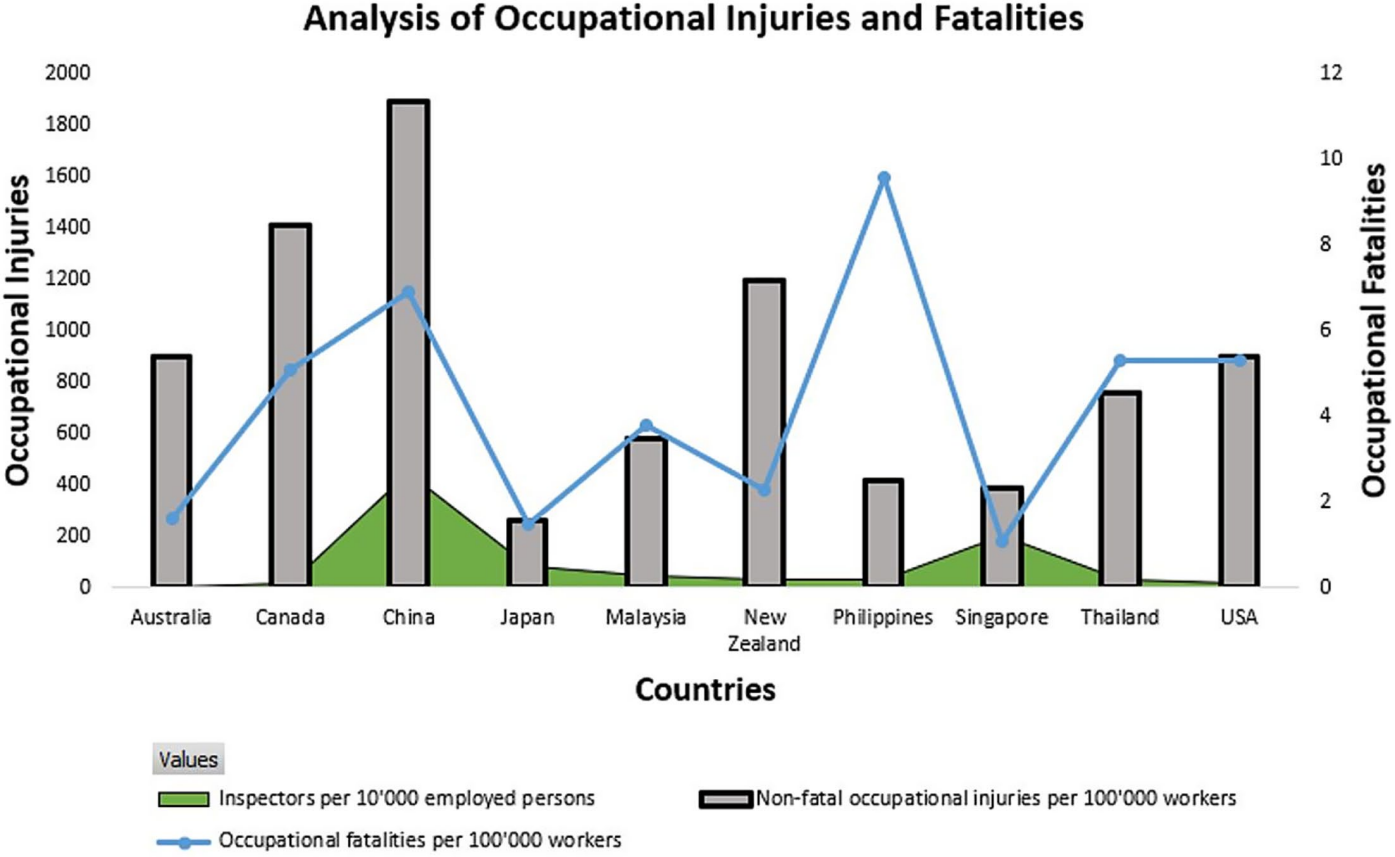
Comparative analysis of different countries by occupational injury and fatality rates (71).

Despite advances in occupational safety regulation and industry standards, the Malaysian O&G sector, particularly its downstream sector continuously faces persistent safety challenges. Characterized by high-risk processing environments, aging infrastructure, and a multi-ethnic workforce, this sector remains vulnerable to both procedural lapses and systemic communication breakdowns. Recent data reveal that while global occupational fatality rates have improved (Figure 1), Malaysia’s downstream O&G operations lag, with incident frequencies surpassing regional benchmarks (12). Human error, often symptomatic of deeper organizational issues, remain a dominant cause exacerbated by weak SCULT, limited safety knowledge, and inconsistent communication practices (13, 14).

Malaysia’s regulatory landscape is shaped by the 1984 Petroleum Act, the 1967 Factory and Machinery Act, and the 1994 OSHA. However, the sector still struggles with effective enforcement, especially in downstream operations, where onshore and offshore challenges differ significantly. According to Nawaz et al. (1) and Sanchez-Gomez et al. (5), broader macroeconomic forces such as financial downturns, pandemic-driven budget constraints, and energy market volatility further compromise organizational commitment to safety investments and training. These external pressures often lead to reprioritization of safety expenditures in favor of operational throughput. In response to these multifactorial risks, there is growing academic and industry consensus on the need to move beyond compliance-based approaches toward systems that embed safety into organizational culture. Studies across different regions reinforce that cultivating a robust SCULT built on trust, leadership visibility, and participatory communication is essential for improving safety outcomes in high-risk industries (14–18). In parallel, SC is increasingly recognized not merely as a function of information dissemination, but as a social and behavioral construct shaped by trust, clarity, timeliness, and feedback loops. CS and employee involvement in safety dialogs substantially enhance hazard recognition and risk mitigation (19, 20). However, SC in many Malaysian O&G settings remains hierarchical and compliance-driven, undermining its effectiveness in engaging frontline workers.

Hazard Identification and Risk Assessment (HIRA) continues to serve as a foundational practice for mitigating site-level risks. Yet, emerging research recommends integrating HIRA into broader organizational learning systems to ensure that risk data is not only recorded but also translated into preventive actions and workforce knowledge (21, 22). The absence of such integration often results in repeated near-miss events and minimal learning from past incidents. Additionally, limited employee involvement in HIRA processes impedes ownership, situational awareness, and safety-related decision-making (23).

This study integrates three core constructs OHSM, SC, and HIRA within a unified safety performance model mediated by SCULT. Rooted in SET, the framework views safety as a reciprocal process: when organizations invest in inclusive safety systems, transparent communication, and trust-building practices, employees reciprocate through knowledge sharing, compliance, and proactive safety behavior (24, 25). Notably, previous studies have examined OHSM, HIRA, and SC in isolation, failing to account for their synergistic effects within a cultural framework. Moreover, the mediating role of SCULT in converting these systemic inputs into improved Safety Knowledge (SK) and Safety Performance (SP) remains underexplored, particularly in the Malaysian downstream O&G context. As such, this study aims to fill that gap by empirically testing a structural model that links these constructs, while integrating contextual realities such as hierarchical communication, economic uncertainty, and workforce diversity. This research contributes to both theory and practice. It provides empirical evidence supporting the role of cultural enablers in safety systems and informs targeted interventions for improving hazard control, communication structures, and workforce engagement in high-risk environments.

### 2.1 Conceptual framework and hypotheses formation

Contemporary literature increasingly highlights the pivotal role of integrated safety management practices in enhancing organizational performance and resilience, particularly in high-risk industries such as oil and gas. SSP has emerged as a strategic imperative, not only for regulatory compliance but also for ensuring operational continuity, environmental stewardship, and workforce well-being. Key components of SSP include structured OHSM, comprehensive HIRA, effective SC, and a strong SCULT. Together, these elements contribute to improve SK among employees and better SP across operations (26, 27).

While previous research has explored these components individually, there is a dearth of studies that empirically examine their interrelationships within a unified framework, particularly in the downstream O&G context. Moreover, few models integrate the mediating role of SCULT or address how these constructs jointly influence knowledge transfer and safety behavior on the ground. Drawing on the principles of SET, this study posits that workplace safety is a reciprocal process, wherein management’s commitment to transparent communication, proactive risk management, and participatory safety systems enhances employee trust, knowledge retention, and compliance with safety protocols. This framework is grounded in SET which suggests that when organizations invest in fair, participatory, and transparent practices, employees reciprocate through enhanced engagement, trust, and compliance. Within the safety domain, this translates into proactive safety behavior, willingness to share safety concerns, and improved hazard responsiveness.

**Figure 2:**
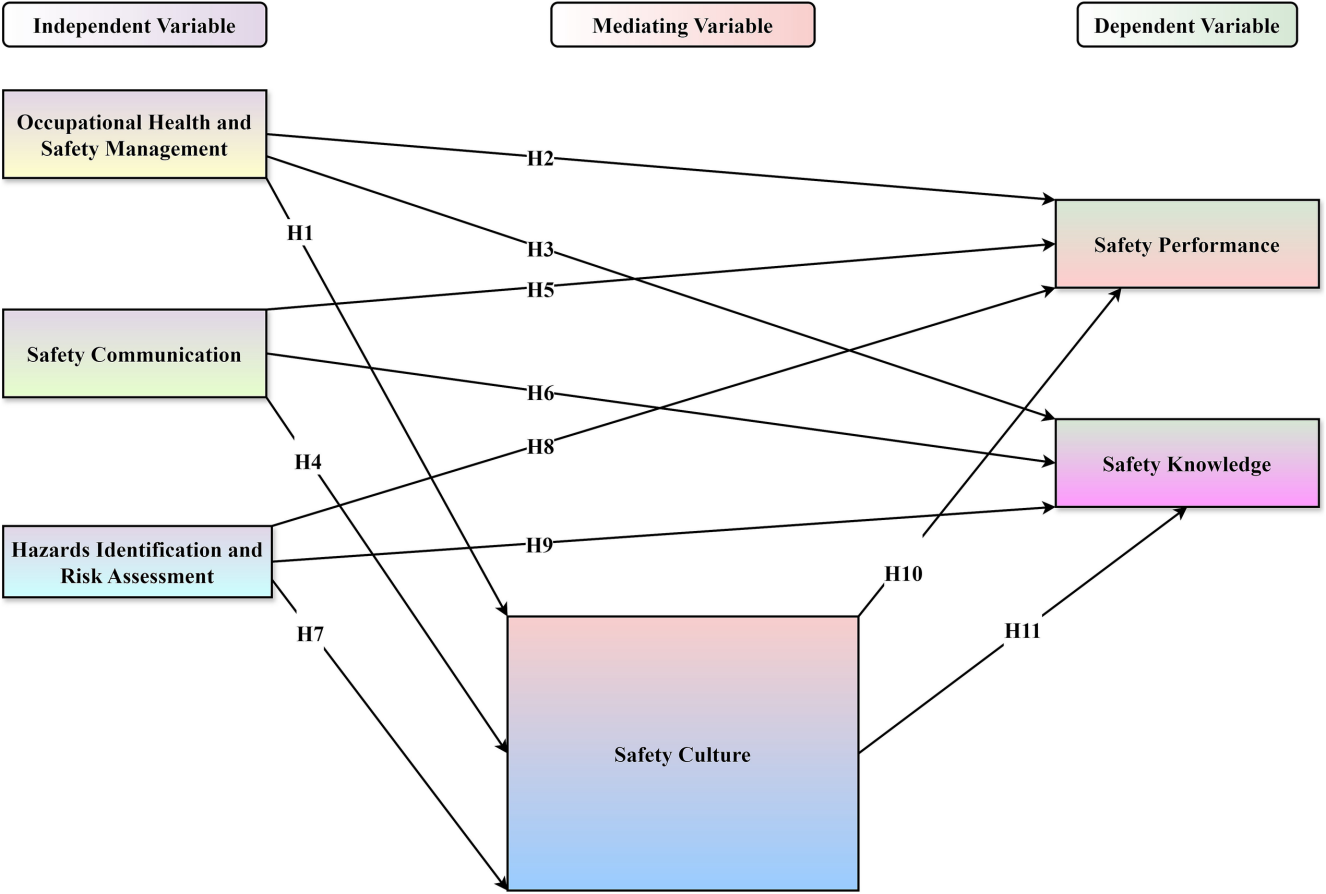
Structural model with hypothesis.

The conceptual framework underpinning this study is adapted from the logic of the Balanced Scorecard (BSC). BSC is a well-established performance measurement system adopted by over 70% of global industries (28, 29). While the BSC traditionally focuses on financial, customer, internal process, and learning perspectives, this study adapts its structure to suit the petrochemical and O&G sectors by emphasizing health and safety as critical performance dimensions. Figure 2 illustrates the proposed framework, which maps the relationships between OHSM, SC, HIRA, SCULT, SK, and SP.

#### 2.1.1 Occupational health and safety management system

OHSM refers to the set of organizational strategies, policies, procedures, and leadership behaviors aimed at promoting workplace health and safety. In high-risk industries, its implementation requires more than compliance. It demands cultural alignment, leadership commitment, and continuous workforce engagement. Despite its recognized value, OHSM implementation often suffers from fragmented leadership attention, constrained resources, and the prioritization of production targets over safety concerns (30). These barriers are especially pronounced in downstream O&G operations, where fast-paced production and complex processing environments can marginalize safety initiatives.

Evidence suggests that effective OHSM enhances employee perceptions of organizational safety commitment, which in turn improves safety behavior and reduces accident rates (31). This study adopts a holistic view of OHSM, emphasizing its alignment with proactive HIRA practices, access to protective equipment, real-time training, and inclusive safety participation. When safety policies are consistently implemented and communicated, they strengthen SCULT, advance knowledge dissemination, and reinforce safety behavior (32). Thus, the hypotheses are:

*Hypothesis* 3: OHSM positively influences SCULT.

*Hypothesis* 3: OHSM has a direct positive impact on SP.

*Hypothesis* 3: OHSM positively enhances SK.

#### 2.1.2 Safety communication

SC is the mechanism through which safety-related information, expectations, and concerns are exchanged within an organization. It plays a critical role in encouraging a shared understanding of risks, aligning safety expectations, and facilitating immediate responses to emerging hazards. Effective SC is characterized by clarity, timeliness, openness, and bidirectional flow. Studies show that upward communication, where employees feel safe voicing concerns, significantly enhances hazard awareness and contributes to injury prevention (33, 34). SC also serves as an indicator of leadership commitment. When leaders actively participate in safety dialogs and model best practices, they legitimize safety priorities and instill confidence across teams (35). Conversely, attributing safety breakdowns solely to worker error, without addressing managerial communication failures, undermines employee trust and weakens SCULT.

In the Malaysian O&G context, nuanced findings have emerged. Even in environments with a generally positive SCULT, operational performance pressures and job security fears may lead employees to deprioritize safety concerns in favor of meeting production targets (36, 37). This stresses the need for structured, consistent SC practices that address both formal and informal communication pathways. Thus, the study’s hypotheses for SC are:

*Hypothesis* 4: SC positively affects SCULT.

*Hypothesis* 5: SC directly improves SP.

*Hypothesis* 6: SC positively influences SK.

#### 2.1.3 Hazard identification and risk assessment

HIRA is a foundational process within OHSMS that systematically identifies, evaluates, and prioritizes potential hazards associated with work activities. Its objective is to develop control measures that mitigate health, safety, and environmental risks before they materialize. Effective HIRA not only reduces operational vulnerability but also cultivates a culture of vigilance and shared responsibility (25). A well-structured HIRA process should be integrated into the local operating management system (LOMS), with clear documentation of procedures, assignment of roles, and training protocols. Active involvement of personnel in hazard assessment promotes ownership and enhances safety awareness. Moreover, regular review of HIRA output ensures alignment with evolving operational conditions and workforce competencies. The value of HIRA extends beyond procedural compliance; it supports SCULT development, improves SP, and builds institutional knowledge that strengthens long-term resilience (24). The proposed hypotheses for HIRA are:

*Hypothesis* 7: HIRA positively affects SCULT.

*Hypothesis* 8: HIRA significantly improves SP.

*Hypothesis* 9: HIRA positively enhances SK

#### 2.1.4 Safety culture

SCULT encompasses the collective values, beliefs, and behaviors that determine how safety is prioritized, communicated, and enacted within an organization. It reflects the depth of leadership commitment to safety, the clarity of policies, and the alignment of individual behavior with organizational goals (38). Empirical evidence associates strong SCULT with lower incident rates, increased employee engagement, and higher compliance with safety procedures (39). As a multidimensional construct, SCULT not only influences SP and SK directly but also mediates the relationship between managerial practices and safety outcomes. Turner et al. emphasized that SCULT emerges from the integration of systems, symbols, and behaviors that minimize risks. Regulatory integration of SCULT, as argued by Antonsen et al. (40), can enhance both compliance and performance at institutional levels.

In high-risk sectors like O&G, cultivating SCULT requires more than symbolic gestures; it demands continuous investment in safety leadership, transparent SC, and inclusive safety participation (33, 41). The mediating role of SCULT is particularly relevant in environments where trust and communication gaps may distort the intended effects of safety interventions. Accordingly, this study hypothesizes both direct and mediating roles of SCULT as:

*Hypothesis* 10: SCULT has a positive impact on SP.

*Hypothesis* 11: SCULT has a positive impact on SK.

*Hypothesis* 12: SCULT mediates the relationship between OHSM and SP.

*Hypothesis* 13: SCULT mediates the relationship between OHSM and SK.

*Hypothesis* 14: SCULT mediates the relationship between SC and SP.

*Hypothesis* 15: SCULT mediates the relationship between SC and SK.

*Hypothesis* 16: SCULT mediates the relationship between HIRA and SP.

*Hypothesis* 17: SCULT mediates the relationship between HIRA and SK.

#### 2.1.5 Safety knowledge

SK refers to employees’ understanding of safety procedures, hazard recognition, response protocols, and their ability to apply this knowledge in practice. It is a vital determinant of effective safety behavior and is closely linked to training effectiveness, communication quality, and leadership involvement (42). Accurate and accessible SK fosters confidence, encourages hazard reporting, and improves decision-making in high-pressure situations (43). Despite its importance, knowledge gaps persist in many O&G settings, particularly among contract workers and shift-based personnel (44). This study posits that SC and SCULT significantly shape the depth and application of SK across operational levels.

#### 2.1.6 Safety performance

SP reflects the extent to which organizations achieve desired safety outcomes, such as reduced injury rates, incident-free operations, and proactive safety behavior. It encompasses both leading indicators (e.g., participation, audits, and training compliance) and lagging indicators (e.g., accident rates, near misses) (45). A high level of SP indicates that safety systems are not only in place but are effectively internalized and practiced by the workforce.

Effective SP requires alignment between technical systems, behavioral interventions, and organizational culture. Employee involvement in safety planning and implementation has been shown to significantly enhance SP outcomes (46). Hence, SP is not only a function of management oversight but a collective measure of organizational safety maturity.

## 3 Research methods

**Figure 3:**
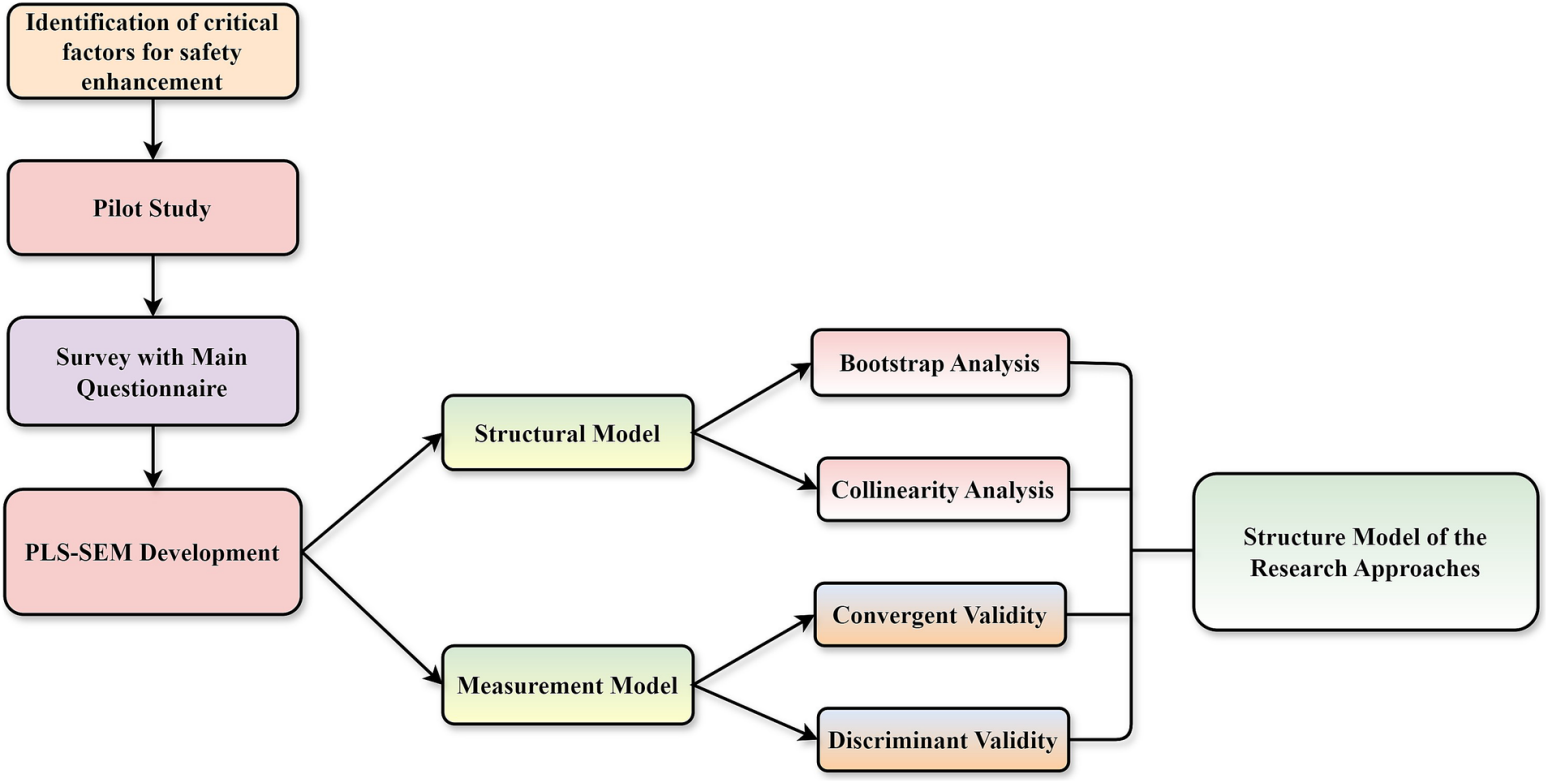
Study’s research flow-chart.

This section outlines the research methodology adopted to investigate the structural relationships between OHSM, SC, HIRA, SCULT, SK, and SP within Malaysia’s downstream O&G industry. A quantitative approach was employed, underpinned by the positivist paradigm, to validate a theoretical model using structural equation modeling techniques. The research design serves as the comprehensive plan of action, encompassing the formulation of research questions, data collection, analysis, and the subsequent discussion and justification (47). This section briefly describes the research method used for this study and Figure 3 presents the research flow chart.

### 3.1 Research design

This study employed a cross-sectional research design, following a quantitative approach. Data was collected at a single point in time, from July to October 2023. This design allowed the study to investigate the relationships between OHSM, SCULT, SC, HIRA, SK, and SP. The study will discuss the details of research methodology in subsequent sections. The selection of the positivist paradigm was guided by its focus on empirically grounded concepts. This approach enabled to gain a comprehensive understanding of the current state of safety practices within the studied population (9, 48).

#### 3.1.1 Questionnaire design

The research instrument was developed based on an extensive literature review and adapted from validated measurement scales to ensure construct validity. The questionnaire was divided into seven sections: demographics, OHSM, SC, HIRA, SCULT, SK, and SP. A five-point Likert scale ranging from 1 (“strongly disagree”) to 5 (“strongly agree”) was employed to capture respondent perceptions with appropriate granularity. To establish content and face validity, initial drafts of the instrument were reviewed by academic experts in occupational health and safety and industry practitioners from the Malaysian O&G sector. Later, minor revisions were made based on clarity, contextual fit, and language appropriateness. Subsequently, interviews were conducted with four downstream O&G professionals to confirm item relevance and comprehension, further strengthening construct reliability.

This study investigated the relationship between OHSM, SCULT, and employee safety outcomes. Therefore, to capture these concepts, a questionnaire was developed based on a thorough review of relevant literature. Items for SC were drawn from (49), ensuring they accurately reflect the exchange of safety information and procedures between supervisors and employees. OHSM, which refers to the overall safety atmosphere created by management safety practices, was measured using items adapted from Guzman et al. (50) and Ammari et al. (51). To assess SCULT, the questionnaire incorporated items based on the work of Dahl & Kongsvik (52) and Newnam & Goode (53), which explore employee contentment with hazard identification and risk assessment processes. Finally, the construct of SK and SP was operationalized using items informed by research from Ertürk et al. (35); Bray & Williams (54); and Ehiaguina (55). These references address employee dedication to safety practices and procedures, ensuring the questionnaire effectively captures this crucial aspect of SCULT.

#### 3.1.2 Sampling and data collection

A stratified purposive sampling technique was employed to ensure proportional representation across job roles (technicians, HSE officers, engineers, and managerial staff) and departments (safety, operations, maintenance). The study was conducted in collaboration with safety departments at PETRONAS Gas Berhad (PGB) and Malaysia LNG (MLNG). Out of 1,400 distributed questionnaires, 350 were completed and validated, yielding a response rate of 25%. The survey was administered via a hybrid approach. Online questionnaires were distributed through official company email lists and paper-based, in-person questionnaires were completed during toolbox meetings and HSE training sessions for employees without frequent digital access. This multi-modal approach (occurred between July and October 2023) ensured inclusivity across various working environments, including shift-based and remote operational units. The final sample was deemed adequate for partial least squares structural equation modeling (PLS-SEM), as recommended by Hair et al. (56) for models with multiple latent constructs.

#### 3.1.3 Pilot study

A pilot study was conducted prior to full-scale deployment to assess the instrument’s reliability and feasibility. The pilot responses were analyzed for internal consistency, response clarity, and survey duration. Based on the pilot findings ambiguities in terminology were resolved, question sequence was adjusted to improve flow, and a Cronbach’s alpha of above 0.7 was confirmed for all constructs, indicating acceptable reliability.

#### 3.1.4 Analytical approach

The study employed partial least square structural equation modeling (PLS-SEM) and PLSpredict to assess out-of-sample prediction for the model due to the robustness in handling complex models with multiple latent constructs, flexibility with smaller sample sizes, and emphasis on prediction and exploratory modeling. SEM is often used in studies to examine and model workplace safety and ergonomics (57, 58). This study utilizes SEM to analyze the complex relationships between the variables in question. SEM is particularly well-suited for investigating safety-related factors within the Malaysian downstream O&G industry. It goes beyond simply identifying patterns in the data by combining elements of multilevel regression and component analysis. This allows for a more nuanced assessment of how well the proposed model fits the actual data and quantifies the relative importance of each factor (59). In SEM, survey data is interpreted by establishing connections between underlying (latent) variables and the observable measures used to collect data (9). To ensure the stability of the analysis, a sample size of 350 respondents was deemed appropriate for this study (56). Furthermore, bootstrapping was employed within the Smart PLS software to assess the model’s robustness and analyze data from the main questionnaire. This technique provides a valuable tool for examining the functionality of the relationships identified through SEM.

## 4 Result

This section presents the results of the data analysis conducted using PLS-SEM. The analysis was performed in two major stages: (1) evaluation of the measurement model and (2) assessment of the structural model, including hypothesis testing. A total of 350 valid responses were included in the analysis, representing various roles across Malaysia’s downstream oil and gas (O&G) operations.

### 4.1 Construct metrics: descriptive and validity analysis

Focusing on O&G downstream accidents in Malaysia and SC’s role in preventing them, this study surveyed 1,400 individuals however 350 individuals responded (87.5% response rate), which yielded five main components: OHSM (18.25%), SC (9.93%), SCULT (7.87%), HIRA (6.56%), SK (6.57%), and SP (3.34%). This explains 38% of the variance, suggesting several factors contributing to safety.

### 4.2 Demographic profile

**Figure 4:**
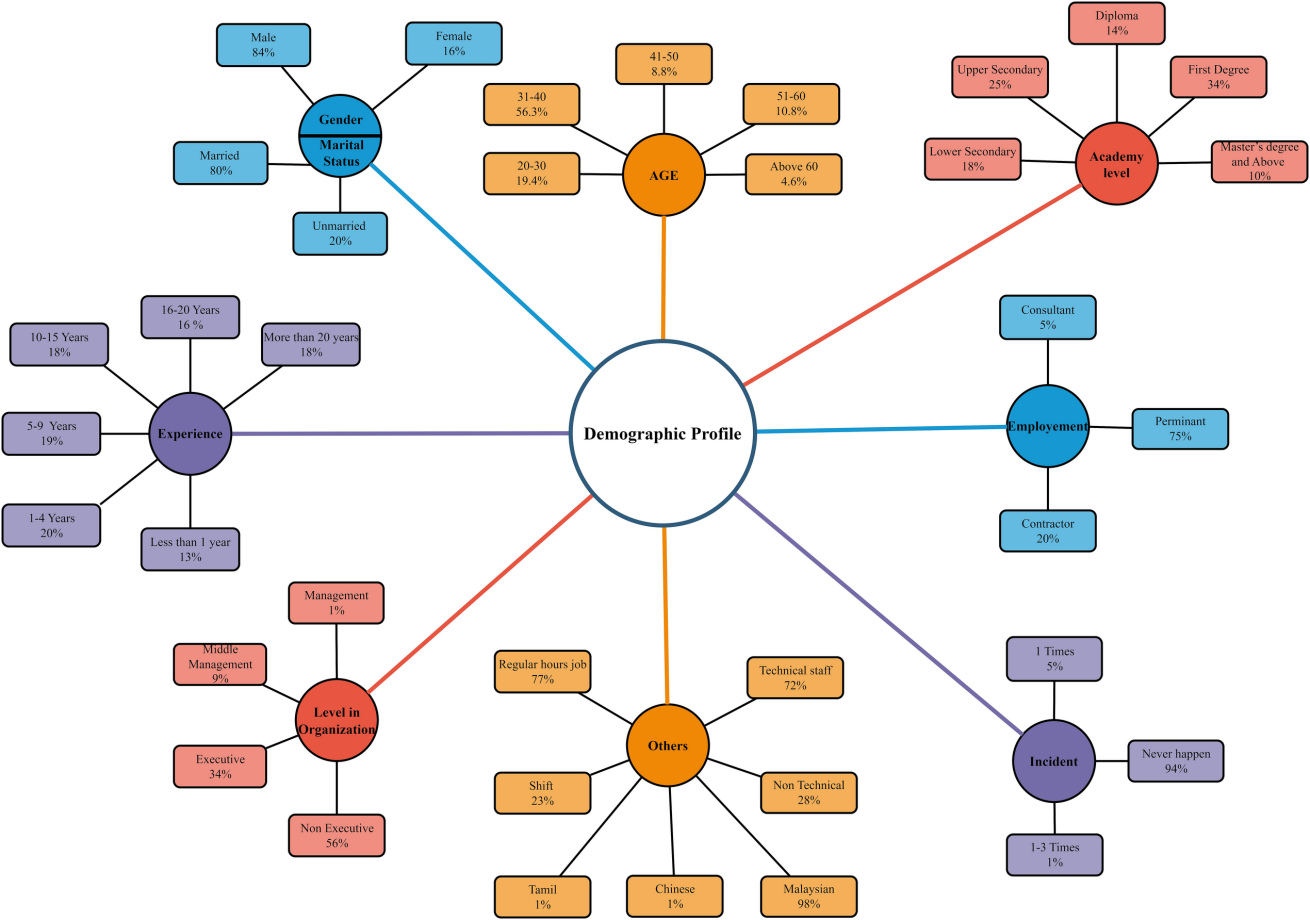
Demographic profile.

The sample consisted of 326 male respondents and 24 females, which aligns with the male-dominated nature of the field. The 68 respondents were aged between 20 and 30, 197 were aged between 31 and 40, 31 were aged between 41 and 50, and 54 respondents were over 51, indicating a relatively youthful and energetic workforce in the PETRONAS O&G industry. Among the participants, 39 were unmarried, while 311 were married (presented in Figure 4). The research participants generally agreed upon the survey items, as indicated by the high mean scores averaging 4 for each construct. The standard deviation (SD) values ranged from 0.420 (for SK1) to 0.803 (for SC8), as reported in Table 1.

**Table 1:** Reliability and validity test outcomes of constructs.

Safety phase	Assigned code	Mean	SD	Initial loadings	Modified	Cronbach alpha	Composite reliability	AVE
Occupational health and safety management (OHSM)	OHSM 1	4.46	0.553	0.813	0.856	0.813	0.864	0.519
	OHSM 2	4.37	0.634	0.798	0.810			
	OHSM 3	4.38	0.707	0.773	0.804			
	OHSM 4	4.12	0.777	0.608	0.672			
	OHSM 5	4.41	0.727	0.789	0.83			
	OHSM 6	4.33	0.641	0.603	0.666			
	OHSM 7	4.31	0.72	0.449	Deleted			
	OHSM 8	3.24	1.305	0.533	Deleted			
Hazard identification and risk assessment (HIRA)	HIRA 1	4.50	0.544	0.801	0.809	0.726	0.829	0.550
	HIRA 2	4.19	0.813	0.645	0.655			
	HIRA 3	4.33	0.694	0.796	0.802			
	HIRA 4	4.32	0.668	0.678	0.686			
	HIRA 5	4.31	0.995	0.789	0.835			
Safety Culture (SCULT)	S_CULT_ 1	4.29	0.713	0.812	0.866	0.885	0.878	0.522
	S_CULT_ 2	3.92	0.981	0.686	0.771			
	S_CULT_ 3	4.27	0.695	0.849	0.894			
	S_CULT_ 4	4.49	0.665	0.788	0.822			
	S_CULT_ 5			0.512	659			
Safety Communication (SC)	SC 1	4.21	0.692	0.699	0.831	0.887	0.912	0.759
	SC 2	4.22	0.742	0.802	0.814			
	SC 3	3.93	0.767	0.791	0.804			
	SC 4	3.97	0.878	0.799	0.805			
	SC 5	4.24	0.717	0.723	0.757			
	SC 6	4.11	0.803	0.551	Deleted			
Safety Knowledge (SK)	SK1	4.8	0.420	0.812	0.822	0.800	0.861	0.555
	SK2	4.55	0.605	0.771	0.805			
	SK3	4.48	0.863	0.533	0.655			
	SK4	4.62	0.535	0.715	0.734			
	SK5	4.43	0.598	0.665	0.696			
Safety Performance (SP)	SP1	4.43	0.598	0.745	0.788	0.903	0.927	0.719
	SP2	4.52	0.544	0.819	0.858			
	SP3	4.24	0.7	0.812	0.817			
	SP4	4.38	0.841	0.874	0.877			
	SP5	4.46	0.907	0.833	0.854			
	SP6	4.53	0.681	0.422	Deleted			
	SP7			0.110	Deleted			

### 4.3 Model assessment

Analyzing the constructs (OHSM and SK/ SP) revealed that numerous items demonstrated statistically significant loading values exceeding 0.5, indicating their strong contribution. However, a closer examination identified five items that exhibit both insignificant contributions and loading values below 0.5, constituting less than 20% of total items, hence excluded. Furthermore, the variance inflation factor (VIF) for the remaining items persisted below 3.3, confirming the absence of multicollinearity issues within the model and that other factors do not inflate the constructs. Finally, subsequent validity and reliability tests confirmed the robustness and validity of the first-order construct.

All measured items in this analysis established internal consistency and reliability criteria. Specifically, all items possessed average variance extracted (AVE) values exceeding 0.5, Cronbach’s alpha surpassing 0.7, and composite reliability exceeding 0.8. These statistics, presented in Table 2, demonstrate that the items consistently measure the intended construct and are free from random error. Notably, while all loading values surpass 0.7, except for item SK5 (0.696), this single instance below the threshold is considered acceptable based on established criteria (56). This indicates that even with this slightly lower loading, the overall internal consistency of the formative construct remains satisfactory.

**Table 2:** Discriminant validity heterotrait-monotrait ratio (HTMT).

Construct	HIRA	OHSM	SP	SCULT	SC	SK
HIRA						
OHSM	0.801					
SP	0.846	0.845				
SCULT	0.674	0.65	0.721			
SC	0.634	0.596	0.647	0.853		
SK	0.835	0.698	0.697	0.534	0.816	

To ensure the entire model’s validity and robustness, this study employed comprehensive assessments focusing on discriminant validity and model fit.

*Discriminant Validity*: This assessment aimed to confirm that the latent constructs within the model are distinct and not simply measuring the same underlying phenomenon. To achieve this, the study examined the correlations between the latent constructs and compared them to the square root of AVE for each construct. If the correlations remain lower than the square root of AVE, it indicates that the constructs are sufficiently distinct. Additionally, the study employed the Heterotrait-Monotrait (HTMT) ratio procedure, which further confirms discriminant validity when HTMT values are less than 1. The Fornell-Larcker criterion (based on correlations and AVE) and the HTMT procedure confirmed discriminant validity in this model. Table 2 highlights the advantages of HTMT over the traditional Fornell-Larcker criterion (presented in Table 3), demonstrating its superiority in discerning truly distinct constructs. The first stage of analysis of formative construct is presented in Table 4.

**Table 3:** Discriminant validity-Fornell-Lacker criterion.

Construct	HIRA	OHSM	SP	SCULT	SC	SK
HIRA	0.761					
OHSM	0.654	0.716				
SP	0.729	0.686	0.768			
SCULT	0.566	0.522	0.616	0.714		
SC	0.563	0.507	0.577	0.756	0.846	
SK	0.727	0.591	0.622	0.466	0.48	0.795

**Table 4:** Latent variables description.

Construct	Items	Outer weight	STDEV	T-value	*p*-value	Outer loading	VIF
Occupational Health and Safety Management (OHSM)	OHSM 1	0.296	0.020	14.914	0.001	0.856	2.366
	OHSM 2	0.291	0.025	11.854	0.001	0.810	1.998
	OHSM 3	0.201	0.021	9.356	0.001	0.738	1.434
	OHSM 4	0.160	0.022	7.228	0.001	0.631	1.463
	OHSM 5	0.213	0.024	8.709	0.001	0.830	1.464
	OHSM 6	0.203	0.021	9.652	0.001	0.717	1.441
	OHSM 7	0.205	0.016	11.722	0.001	0.756	1.452
	OHSM 8	Dropped					
Safety Culture (SCULT)	S_CULT_ 1	0.246	0.016	5.777	0.001	0.866	1.577
	S_CULT_ 2	0.247	0.011	22.84	0.001	0.894	3.450
	S_CULT_ 3	0.231	0.021	18.334	0.001	0.822	2.278
	S_CULT_ 4	0.222	0.012	14.136	0.001	0.729	1.482
	S_CULT_ 5	0.163	0.013	16.559	0.001	0.771	1.802
Safety Communication (SC)	SC 1	0.237	0.013	18.803	0.001	0.740	2.598
	SC 2	0.191	0.016	12.266	0.001	0.788	2.704
	SC 3	0.201	0.014	14.854	0.001	0.859	2.056
	SC 4	0.210	0.013	14.795	0.001	0.984	2.060
	SC 5	0.192	0.017	14.771	0.001	0.874	1.802
	SC 6	Dropped					
Safety Knowledge (SK)	SK1	0.416	0.019	3.844	0.001	0.854	2.036
	SK2	0.292	0.023	11.298	0.001	0.778	1.463
	SK3	0.348	0.016	5.777		0.877	1.858
	SK4	0.375	0.011	22.844	0.001	0.826	1.692
	SK5	0.282	0.014	23.002	0.001	0.747	1.407
Safety Performance (SP)	SP1	0.239	0.016	15.236	0.001	0.815	2.388
	SP2	0.259	0.015	16.870	0.001	0.836	2.882
	SP3	0.183	0.014	13.274	0.001	0.853	3.511
	SP4	0.220	0.011	20.282	0.001	0.891	1.196
	SP5	0.226	0.014	16.344	0.001	0.834	1.099
	SP6	Dropped					
	SP7	Dropped					
Hazard Identification and Risk Assessment (HIRA)	HIRA 1	0.416	0.026	16.280	0.001	0.809	1.463
	HIRA 2	0.292	0.028	10.557	0.001	0.655	1.239
	HIRA 3	0.348	0.019	18.035	0.001	0.802	1.611
	HIRA 4	0.282	0.025	11.144	0.001	0.86	1.360
	HIRA 5	0.261	0.020	14.914	0.001	0.812	2.366

*Model Fit*: This assessment ensures that the proposed model adequately represents the observed data. This study employed the standardized-root-mean-square-residual (SRMSR) as a key indicator of model fit. An SRMSR value below 0.08 generally suggests a good fit. In this case, the obtained value of 0.078 further confirms that the model accurately reflects the relationship between the constructs and variables. R-squared (R^2^) values for each endogenous construct also provide insights into the model’s explanatory power. Acceptable R^2^ values in this analysis indicate that the model effectively explains a significant portion of the variance in the dependent variables.

*Hypothesis Testing*: Finally, we rigorously tested the 17 proposed hypotheses within the model using a bootstrapping procedure with 5,000 samples. This technique strengthens the reliability of the results by simulating repeated samples and assessing the stability of the findings. The bootstrapping analysis confirmed 11 hypotheses have a significant impact while two hypothesis were rejected, with detailed results in Table 5. Additionally, consistent PLS (PLSc) was employed throughout the analysis to ensure consistent estimation of path coefficients and indicator loadings, particularly relevant for models with non-recursive structures, as outlined by Hair et al. (56).

**Table 5:** Result of hypothesis testing.

Path coefficient	*β*	STDEV	T Statistics	*p*-values
H1 OHSM→SCULT	0.343	0.059	5.829	0.001
H2 OHSM→SK	0.125	0.06	2.093	0.001
H3 OHSM→SP	0.125	0.064	1.946	0.001
H4 SC→SCULT	0.111	0.059	1.891	0.059
H5 SC→SK	0.053	0.066	0.81	0.418
H6 SC→SP	0.096	0.065	1.468	0.142
H7 HIRA→SCULT	0.424	0.053	7.931	0.001
H8 HIRA→SK	0.361	0.068	5.269	0.001
H9 HIRA→SP	0.347	0.07	4.933	0.001
H10 SCULT→SK	0.203	0.054	3.732	0.001
H11 SCULT→SP	0.272	0.058	4.645	0.001
H12 OHSM→SCULT→SK	0.13	0.035	3.672	0.001
H13 OHSM→SCULT→SP	0.099	0.031	3.184	0.001
H14 SC→SCULT→SK	0.625	0.036	17.371	0.001
H15 SC→SCULT→SK	0.826	0.019	43.981	0.001
H16 HIRA→SCULT→SK	0.301	0.013	23.456	0.001
H17 HIRA→SCULT→SK	0.255	0.015	13.002	0.001
		R^2^	Q^2^	
SCULT		0.571	0.354	
SK		0.613	0.189	
SP		0.417	0.229	
OHSM		0.387	0.354	

#### 4.3.1 OHSM

The results strongly support the positive influence of OHSM on all three key outcomes. In H1 OHSM demonstrated a significant impact on SCULT, revealing a significant association (H1: *β* = 0.343, *t* = 5.829, *p* < 0.001), SK (H2: *β* = 0.125, *t* = 2.093, *p* < 0.001), and safety performance (H3: *β* = 0.125, *t* = 1.946, *p* < 0.001). These findings reinforce the previous assertions that effective OHSM systems create a structured safety environments that strengthens organizational learning, reduces hazard exposure, and ensures behavioral compliance (43). Recognizing its significance, Alcantara et al. (60) assert that OHSM is vital for promoting SK/performance and ensuring employees receive essential education and information. OHSM’s effect on SCULT aligns with the principles of SET, indicating that when organizations visibly prioritize safety, employees are more likely to engage in reciprocal safety behaviors. This reflects the cultural embedding of safety protocols into daily operations, which translates into improved awareness and performance.

#### 4.3.2 SC

The results reveal a more nuanced role of SC. While SC had a marginally significant effect on SCULT (H4: *β* = 0.111, t = 1.891, *p* = 0.059), its direct relationships with SK (H5: *β* = 0.053, *t* = 0.810, *p* = 0.418) and SP (H6: *β* = 0.096, *t* = 1.468, *p* = 0.142) were statistically nonsignificant. This suggests that SC, when practiced as a unidirectional or compliance-driven tool, may lack the capacity to influence knowledge retention and behavioral change independently. However, its marginal impact on SCULT hints at a latent influence, emphasizing the importance of communication quality, openness, and consistency. These findings resonate with earlier literature, including Fernández-Muñiz et al. (61); Neal & Griffin (62); and Xia et al. (63), which advocates that SC should be embedded in both regulatory systems and organizational norms to improve hazard awareness and behavioral commitment.

#### 4.3.3 Hira

HIRA emerged as a strong predictor of all three safety outcomes. It significantly influenced SCULT (H7: *β* = 0.424, t = 7.931, *p* < 0.001), SK (H8: *β* = 0.361, *t* = 5.269, *p* < 0.001), and SP (H9: *β* = 0.347, *t* = 4.933, *p* < 0.001). These results validate the central role of structured risk assessments in high-risk industries like oil and gas. The findings indicate that when HIRA is implemented with clear accountability and ongoing feedback mechanisms, it strengthens situational awareness and creates a culture of shared responsibility. This aligns with research by Iqbal et al. (25), which emphasizes the capacity of HIRA to transform reactive safety postures into proactive hazard prevention strategies.

#### 4.3.4 Safety culture

Safety culture (SCULT) demonstrated a robust influence on both SK and SP. The results confirm H10 (*β* = 0.203, *t* = 3.732, *p* < 0.001) and H11 (*β* = 0.272, *t* = 4.645, *p* < 0.001), indicating that SCULT is not only a product of organizational systems but also a key driver of behavioral and cognitive safety outcomes.

Furthermore, mediation analysis revealed that SCULT plays a significant intermediary role across multiple paths:

- H12: OHSM → SCULT → SK (*β* = 0.130, *t* = 3.672, *p* < 0.001)
- H13: OHSM → SCULT → SP (*β* = 0.099, *t* = 3.184, *p* < 0.001)
- H14: SC → SCULT → SK (*β* = 0.625, *t* = 17.371, *p* < 0.001)
- H15: SC → SCULT → SP (*β* = 0.826, *t* = 43.981, *p* < 0.001)
- H16: HIRA → SCULT → SK (*β* = 0.301, *t* = 23.456, *p* < 0.001)
- H17: HIRA → SCULT → SP (*β* = 0.255, *t* = 13.002, *p* < 0.001)

These mediation results validate the central theoretical proposition of the study: that SCULT serves as the organizational climate through which formal systems (OHSM, SC, HIRA) manifest their influence on safety outcomes. In accordance with SET, it reflects the reciprocity between management actions and employee safety engagement. These findings align with the work of Zhao et al. (64) and Wang & Bielicki (65), emphasizing the significant impact of SCULT on hazard recognition and prevention in organizations. Thus, maintaining up-to-date knowledge through effective practices is a crucial control tool for accident and injury prevention.

### 4.4 Mediating latent effects

This section explores the indirect effects of OHSM, SC, and HIRA on SP and SK through the mediating role of SCULT. The analysis, presented in Tables 5, 6, reveals that SCULT significantly mediates multiple structural relationships, reinforcing its pivotal function in facilitating sustainable safety outcomes in the downstream oil and gas (O&G) sector.

**Table 6:** Summary view of hypotheses strengths.

Category	Hypotheses
Strongly supported	H14, H15
Supported	H1, H2, H3, H7, H8, H9, H10, H11, H12, H13, H16, H17
Partially/marginally supported	H4
Not Supported	H5, H6

The mediating effect of SCULT between OHSM and both SP and SK is statistically significant. Specifically, the indirect path from OHSM to SK via SCULT (H12: *β* = 0.130, *t* = 3.672, *p* < 0.001) and from OHSM to SP via SCULT (H13: *β* = 0.099, *t* = 3.184, *p* < 0.001) confirms that cultural alignment is essential for translating safety management systems into practical safety gains. While OHSM had its own direct effects on both SK and SP, the added significance of its indirect effects underlines the reinforcing role of a safety-oriented work climate.

Similarly, HIRA demonstrated strong mediated effects on both SK and SP through SCULT. The results confirm the mediation paths (H16: *β* = 0.301, *t* = 23.456, *p* < 0.001; H17: *β* = 0.255, *t* = 13.002, *p* < 0.001), indicating that structured risk assessment protocols do not act in isolation but are most effective when embedded in a culture of trust, shared responsibility, and continuous learning. These findings reinforce earlier research suggesting that HIRA’s success is amplified by strong SCULT and responsive leadership (25).

Notably, while the direct impact of SC on SK and performance was statistically insignificant (H5 and H6), its indirect effects through SCULT were among the strongest in the model. SC exhibited a robust mediated impact on SK (H14: *β* = 0.625, *t* = 17.371, *p* < 0.001) and an even stronger indirect influence on SP (H15: *β* = 0.826, *t* = 43.981, *p* < 0.001). These findings imply that communication in the O&G context may be ineffective unless it is trusted, culturally grounded, and participatory. The results align with studies emphasizing the need to move from compliance-based SC models to interactive, socially embedded communication systems (66).

In contrast, total effects for SC on SP and SK (including both direct and indirect pathways) remain statistically marginal or non-significant when cultural mediation is weak or absent. For example, SC → SP (T = 0.909, *p* = 0.363) and SC → SK (T = 1.719, *p* = 0.086) without SCULT mediation fail to reach conventional thresholds. These results suggest that SC alone is insufficient in driving behavioral change unless accompanied by cultural transformation.

Overall, these findings affirm that SCULT is not merely a background factor, but a central latent construct that enables or inhibits the effectiveness of organizational safety systems. Organizations must therefore cultivate SCULT proactively if they wish to realize the full benefits of technical interventions such as OHSM, SC, and HIRA. This requires aligning communication practices, leadership engagement, and hazard management strategies within a coherent cultural framework to maximize impact on SK and performance (66).

### 4.5 Discussion

This study offers a multidimensional view of how safety practices and outcomes are shaped within high-risk operational environments. Drawing on SET, the results confirm that formal safety systems (OHSM, HIRA, and SC) only translate into meaningful SK and SP when mediated through a strong SCULT. One of the most revealing findings is the behavior of SC. Although SC is commonly positioned in the literature as a pivotal enabler of safety outcomes, the empirical findings demonstrate that SC does not exert a statistically significant direct effect on either Safety Knowledge (SK) or Safety Performance (SP), as reflected in the results for H5 and H6. This challenges traditional assumptions that view communication as an isolated lever of behavioral change. In complex, hierarchical environments such as Malaysia’s downstream O&G sector, where communication often follows a top-down format, SC alone may lack the relational depth, contextual relevance, or psychological safety necessary to directly influence frontline behavior or cognitive safety awareness (33, 39).

However, this does not negate the strategic value of SC. Rather, its true influence is realized when embedded within a strong SCULT. The significant indirect effects of SC mediated through SCULT (H14, H15) highlight that communication becomes effective when grounded in a culture of mutual trust, fairness, and openness. This finding reinforces the premise of SET, which asserts that individuals are more likely to internalize safety messaging and reciprocate safe behavior when they perceive organizational sincerity and fairness. Thus, SC functions not as a standalone mechanism, but as a relational facilitator whose efficacy is contingent on the cultural ecosystem in which it operates. Furthermore, SCULT’s mediating role was not limited to SC alone but it significantly influenced the transmission of OHSM and HIRA effects as well. This affirms SCULT as the trust mechanism that transmits institutional intent into employee engagement (67, 68). For OHSM, the results reveal both direct and indirect pathways toward SK and SP, highlighting the importance of structured systems (e.g., hazard reporting, training, procedural clarity) as foundational, but not sufficient on their own. The effectiveness of OHSM systems is amplified when employees internalize them through a supportive culture that promotes inclusion and accountability.

HIRA emerged as the strongest direct predictor across all dependent variables. Its impact on SK and SP highlights the proactive potential of participatory risk assessment in building situational awareness and behavioral discipline. Employees who are involved in identifying hazards tend to demonstrate stronger cognitive retention and safer decision-making. Moreover, HIRA’s mediated effects through SCULT indicate that the effectiveness of hazard assessments improves when embedded within a trusting culture that fosters shared ownership. This finding aligns with previous research that advocates for dialogic, inclusive, and psychologically safe communication channels in high-risk sectors (13, 66). In essence, it is not just the presence of communication that matters, but its quality, tone, and contextual trustworthiness that determine its effectiveness. The findings substantiate SET in the context of occupational safety. The results illustrate that when organizations fulfill their implicit “contract” with employees by offering safety systems, risk mitigation, and open communication, employees reciprocate with higher engagement, knowledge sharing, and compliance. This reciprocity is particularly evident in the mediating role of SCULT, which functions as a relational bridge between institutional inputs and employee outputs.

Collectively, these findings offer a sector-specific extension of SET, illustrating how both formal structures (OHSM and HIRA) and soft variables (SC and SCULT) must interact to deliver tangible safety outcomes (69). From a practical standpoint, the results advocate investing in SCULT as a strategic asset. Leadership visibility in safety initiatives, transparent communication channels, real-time feedback loops, and workforce empowerment are not peripheral practices but central levers for improving both safety behavior and knowledge (70). For managers, this means evolving from compliance-oriented leadership to relational safety leadership. For policymakers, regulatory frameworks such as Malaysia’s OSHA should consider incorporating leading indicators like cultural cohesion, communication satisfaction, and psychological safety, moving beyond retrospective incident tracking. In summary, the study challenges a one-size-fits-all approach to safety and emphasizes that technical systems only yield results when reinforced by a culture of trust, mutual obligation, and engagement. Safety outcomes are thus best sustained through the co-evolution of structure and culture, formal systems and informal norms.

## 5 Conclusion

This study offers a comprehensive examination of sustainable safety practices in Malaysia’s downstream O&G industry by exploring the interconnected roles of OHSM, HIRA, and SC, with SCULT acting as a mediating construct. Through an empirical investigation involving 350 respondents across PETRONAS-affiliated downstream sites, the research employs PLS-SEM to validate a multidimensional safety framework grounded in SET. The findings reveal that while OHSM and HIRA significantly and directly influence both SK and SP, the direct effects of SC were not statistically supported. This challenges longstanding assumptions in the safety literature that position communication as a direct determinant of safety outcomes. Instead, the analysis confirms that SC demonstrates substantial influence only when transmitted through a trust-anchored and participatory SCULT. SCULT not only mediates SC’s effects but also enhances the impact of OHSM and HIRA acting as a relational bridge that translates structural inputs into behavioral and cognitive outcomes.

The study advances theoretical understanding by empirically demonstrating that cultural embedding is essential for converting managerial systems into sustained workforce engagement. From a practical perspective, it offers industry-specific recommendations, including the development of real-time digital hazard reporting tools, behavior-based safety (BBS) training, and leadership development programs that prioritize communication satisfaction and cultural alignment. These tools can reinforce proactive hazard recognition, build trust in communication processes, and elevate accountability across all organizational levels. In terms of contextual relevance, the proposed framework is particularly well-suited for high-density, multicultural, and operationally complex environments such as those found in the downstream oil and gas sector. It accommodates variable communication needs and hierarchical structures, making it a flexible yet robust model for cross-functional safety improvement. While not predictive in a strict statistical sense, its applied utility provides a foundation for ongoing diagnostics, risk assessment refinement, and cultural monitoring.

Finally, the study lays the groundwork for future exploration. Longitudinal and mixed-methods research could assess how safety culture evolves under shifting organizational and economic conditions. Additionally, expanding the framework to incorporate sustainability indicators such as environmental performance and corporate social responsibility would provide a more holistic understanding of sustainable safety management in volatile and high-risk industries.

## Data Availability

The raw data supporting the conclusions of this article will be made available by the authors, without undue reservation.
